# Contralateral ovarian endometrioma recurrence after unilateral salpingo-oophorectomy

**DOI:** 10.1186/s12905-019-0760-z

**Published:** 2019-05-02

**Authors:** Tokie Hidari, Tetsuya Hirata, Tomoko Arakawa, Kaori Koga, Kazuaki Neriishi, Shinya Fukuda, Akari Nakazawa, Natsuki Nagashima, Suke Ma, Hui Sun, Masashi Takamura, Miyuki Harada, Yasushi Hirota, Osamu Wada-Hiraike, Tomoyuki Fujii, Yutaka Osuga

**Affiliations:** Department of Obstetrics and Gynecology, Faculty of Medicine, University of Tokyo, 7-3-1 Hongo, Bunkyo-ku, Tokyo, 113-8655 Japan

**Keywords:** Endometriosis, Recurrence, Unilateral endometrioma, Unilateral salpingo-oophorectomy

## Abstract

**Background:**

The recurrence rate after unilateral salpingo-oophorectomy (USO) for unilateral endometrioma has not been reported. We evaluated the rate of and risk factors for endometrioma recurrence after USO.

**Methods:**

In this retrospective observational study, we enrolled 110 women (age, 35–45 years) who underwent laparoscopic USO (*n* = 50) or cystectomy (*n* = 60) for unilateral ovarian endometrioma from January 2010 through December 2012. We compared patients’ characteristics between patients who underwent USO and those who underwent cystectomy. We also compared patients with and without an endometrioma recurrence after USO using univariate and multivariate stepwise logistic regression models to identify recurrence risk factors. Endometrioma recurrence was defined as an ovarian cyst (> 2 cm) with features typical of an endometrioma identified by postoperative transvaginal sonography.

**Results:**

Endometrioma recurred in 8 (16%) patients after USO (mean follow-up, 46.0 ± 12.9 months [range, 15–73]). The post-USO cumulative recurrence rates at 12, 24, 36, and 60 months were 8.0, 10.2, 12.7, and 24.7%, respectively (Kaplan-Meier analysis). In logistic regression analysis, a contralateral side adhesion score ≥ 4 was an independent risk factor for endometrioma recurrence after USO (odds ratio, 19.48, 95% confidence interval, 1.59–237.72). The post-USO cumulative recurrence rates at 12, 24, 36, and 57 months were 19.5, 24.1, 31.0, and 54.0%, respectively, in cases with contralateral side adhesion scores ≥4, and 0.0, 0.0, 0.0, and 5.9%, respectively, in cases with scores < 4 (log-rank test, *P* = 0.0023).

**Conclusions:**

To our knowledge, this is the first report on the recurrence rate and risk factors associated with recurrence after USO. Endometrioma recurrence rates were 24.7% during the first 5 years after USO. The post-USO recurrence rate increased significantly in cases with contralateral side adhesions. Our findings could improve the planning of USO and patient selection for postoperative hormonal therapy.

## Background

Endometriosis is a chronic inflammatory disease caused by the development of tissues resembling endometrium outside the uterus, mainly in the ovary, uterosacral ligament, and pelvic peritoneum [1–3]. Endometriosis affects approximately 10% of women in their reproductive years and is associated with dysmenorrhea, pelvic pain, and infertility. Ovarian endometriomas are reportedly present in 17–44% of endometriosis cases [4]. Laparoscopic surgery should be considered for patients who require pain relief, cyst removal, or histological diagnosis, although the risk of diminishing ovarian reserve should also be considered [1].

Typically, conservative surgery is preferred over radical procedures because most patients with endometrioma are of the reproductive age [5]. However, a systematic literature review estimated the 2- and 5-year endometriosis recurrence rates after conservative surgery as 21.5% and 40–50%, respectively [6]. Furthermore, the recurrence rate after a second surgery may be comparable to that after the first conservative surgery for endometrioma [7]. Oophorectomy is sometimes performed as a semi-radical surgery when the ovarian cyst is large, multiloculated, or potentially malignant. Unilateral oophorectomy may be well-suited as a radical treatment for unilateral ovarian endometrioma in women who do not plan to conceive in the future with the expectation that the risk of postoperative recurrence will be reduced. According to the largest study on the incidence of premenopausal unilateral oophorectomy, 295/1838 (20.1%) patients who underwent the procedure for a medical indication were pathologically diagnosed with endometriosis [8].

However, there are no reports on the disease recurrence rate after USO for unilateral endometrioma. Therefore, we investigated the rate of and risk factors for disease recurrence after USO for unilateral ovarian endometrioma. To our knowledge, this is the first report on the recurrence rate and risk factors associated with recurrence after USO.

## Methods

We performed a retrospective study of patients aged 35–45 years who underwent laparoscopic USO or laparoscopic cystectomy for an MRI-confirmed unilateral ovarian endometrioma at the University of Tokyo Hospital between 2010 and 2012.

The inclusion criteria were: age between 35 and 45 years, and preoperative magnetic resonance imaging (MRI)-confirmed unilateral ovarian endometrioma. The exclusion criteria were: the presence of bilateral endometriomas at study enrollment, previous unilateral or bilateral oophorectomy, and pathological diagnosis of malignancy.

We enrolled 110 patients after reviewing the medical records. We recorded the patient age at the time of the operation; history of pregnancy, parturition, dysmenorrhea, and infertility; diameter of the largest cyst; the presence of multilocular cysts, uterine fibroids, and adenomyosis; the revised American Society for Reproductive Medicine (r-ASRM) [9] classification score; r-ASRM ipsilateral and contralateral side adhesion scores; and postoperative hormonal therapy, pregnancy, delivery, follow-up length, and recurrence. According to the r-ASRM score system, the right and left ovary and fallopian tube adhesion scores were summed separately, and we defined the ovarian endometrioma side as ipsilateral. A unilateral ovarian endometrioma was confirmed in all patients by preoperative 3.0 Tesla MRI. One day prior to surgery, transvaginal ultrasound confirmed the presence of endometrioma and the absence of endometrioma on the contralateral side in all cases. Patients were divided into the USO and cystectomy groups after reviewing and comparing medical records. The Institutional Review Board of Tokyo University Hospital approved the study. All patients provided written informed consent to participate in the research before surgery.

### Surgery

Laparoscopic surgery for ovarian endometrioma began with an inspection of the pelvis and ovarian adhesiolysis.

Laparoscopic USO continued with isolation, coagulation, and division of the ovarian artery and vein. The pelvic peritoneal incision was extended toward the ovarian ligament, and the oviduct and ovarian ligament were coagulated and divided using bipolar electrocoagulation. Upon completion of USO, bipolar electrocoagulation was used to obtain hemostasis. Alternatively, laparoscopic ovarian cystectomy continued with a sharp cortical incision and identification of a cleavage plane as previously described [10, 11]. The cyst capsule was completely stripped from the ovarian tissue using appropriate traction and sharp dissection.

In both procedures, all endometriotic peritoneal implants were excised with scissors or coagulated with bipolar electrocoagulation. The surgeon also inspected the contralateral ovary and fallopian tube and performed adhesiolysis as required. Coagulation was used to obtain hemostasis after adhesiolysis. The surgeons assigned r-ASRM scores according to the intraoperative findings [9]. We retrospectively reviewed the surgical records, including r-ASRM scores, of all patients.

### Definition of recurrent ovarian endometrioma

All patients were instructed to undergo transvaginal ultrasound monitoring for recurrence every 3 months postoperatively. We defined endometrioma recurrence as in our previous studies [10–12]. Briefly, recurrence was defined as the presence of an ovarian cyst (> 2 cm) with typical features of an endometrioma identified on ultrasound [13]. A diagnosis of recurrence was made when the cyst was repeatedly observed with ultrasonography or MRI.

### Statistical analysis

We used JMP Pro 14 software (SAS Institute Inc., Cary, NC, US) for all statistical analyses. Continuous data were analyzed with t-tests and are presented as means and standard deviations (SDs). Categorial data were analyzed with chi-square and Fisher’s exact tests and are presented as counts and percentages. Univariate and multivariate stepwise logistic regression analyses were performed to compare the clinical characteristics of patients with and without ovarian endometrioma recurrence. The Kaplan-Meier method and log-rank test were used to compare the postoperative cumulative recurrence rates. A *P*-value of < 0.05 was considered statistically significant.

## Results

### Patient and case characteristics

The demographic variables, intraoperative findings, and postoperative course of all 110 patients with unilateral ovarian endometrioma are shown in Table 1. Compared to the cystectomy group, patients in the USO group were older at the time of surgery; there were also more preoperative pregnancies and births, as well as fewer infertile patients in the USO group. The r-ASRM and ipsilateral side adhesion scores were significantly higher in the USO group than in the cystectomy group.

**Table 1:** Characteristics of patients who underwent USO or cystectomy for unilateral ovarian endometrioma

	USO	cystectomy	*P* value
N	50	60	
Age at the time of operation	41.4 ± 2.3 (36–44)	38.2 ± 2.4 (35–43)	*P* < 0.0001
Gravity	0.86 ± 0.94 (0–3)	0.22 ± 0.53 (0–3)	P < 0.0001
Parity	0.67 ± 0.80 (0–3)	0.17 ± 0.46 (0–2)	P < 0.0001
Right endometrioma	25 (50.0%)	30 (50.0%)	
Left endometrioma	25 (50.0%)	30 (50.0%)	NS
Diameter of largest cyst (cm)	5.64 ± 1.91 (2–10)	5.26 ± 2.65 (1–15)	NS
Multilocular cyst	9 (18.0%)	9 (15.0%)	NS
Leiomyoma	22 (44.0%)	25 (41.7%)	NS
Adenomyosis	8 (16.0%)	9 (15.0%)	NS
Dysmenorhea	29 (59.2%)	24 (40.0%)	NS
Infertility	4 (8.5%)	19 (31.7%)	*P* = 0.0042
Concurrent hysterectomy	2 (4.0%)	0 (0.0%)	NS
reASRMscore	55.5 ± 28.5 (21–126)	43.8 ± 27.6 (16–124)	*P* = 0.0321
Adhesion score in the ipsilateral side	11.5 ± 7.9 (0–32)	8.4 ± 8.2 (0–32)	*P* = 0.0476
Adhesion score in the contralateral side	5.1 ± 8.3 (0–32)	3.8 ± 7.9 (0–32)	NS
Adhesion score in the ipsilateral side ≥4	45 (90.0%)	48 (80.0%)	NS
Adhesion score in the ipsilateral side ≥16	21 (42.0%)	13 (27.7%)	*P* = 0.0245
Adhesion score in the contralateral side ≥4	21 (42.0%)	16 (26.7%)	NS
Adhesion score in the contralateral side ≥16	5 (10.0%)	7 (11.7%)	NS
Pouch of Douglas obliteration	33 (66.0%)	27 (45.0%)	*P* = 0.0349
Postoperative follow-up period(month)	46.0 ± 12.9 (15–73)	38.8 ± 22.0 (4–75)	*P* = 0.0425
Postoperative hormonal therapy	10 (20.0%)	24 (41.7%)	P = 0.0375
Oral contraceptives	3 (6.0%)	16 (26.7%)	
Dienogest	7 (14.0%)	8 (13.3%)	
Postoperative fertility treatment	2 (4.0%)	18 (42.9%)	*P* = 0.0004
Postoperative pregnancy	0 (0.0%)	8 (13.3%)	*P* = 0.0076
Postoperative parturition	0 (0.0%)	7 (11.7%)	*P* = 0.0165
Postoperative recurrence	8 (16.0%)	7 (11.7%)	NS
Time to recurrence (month)	22.3 ± 19.7 (2–54)	17.7 ± 15.6 (2–45)	NS

Postoperative hormonal therapy was administered in 10/50 cases after USO and 24/60 cases after cystectomy (20.0% vs. 41.7%, *P* = 0.0375). Postoperative fertility treatment, pregnancy, and delivery were also less frequent in the USO group than in the cystectomy group. Endometrioma recurrence occurred in 8 (16.0%) and 7 (11.7%) patients in the USO and cystectomy groups, respectively.

### Characteristics of patients with and without recurrence after USO

Table 2 shows the characteristics of cases with and without post-USO recurrence. In univariate analysis, the frequency of patients with a contralateral side adhesion score ≥ 4 was significantly higher in the recurrence group than in the non-recurrence group. The postoperative follow-up period was significantly longer in patients with than in those without a recurrence. We identified largest cyst diameter, r-ASRM score, ipsilateral side adhesion score ≥ 16, and contralateral side adhesion score ≥ 4 as factors for logistic regression analysis using forward stepwise variable selection. Of these, only a contralateral side adhesion score ≥ 4 was independently associated with recurrence (*P* = 0.020; OR, 19.48; 95% CI, 1.59–237.72). Douglas pouch obliteration and r-ASRM score were not associated with the recurrence rate.

**Table 2:** Univariate and logistic regression analysis of factors related recurrence after USO for unilateral ovarian endometrioma

			univariate analysis	logitic regression analysis	
	recurrence(+)	recurrence(−)	P value	P value	OR (95%CI)
N	8	42			
Age at the time of operation	41.0 ± 2.9 (36–44)	41.5 ± 2.2 (36–44)	NS		
Gravity	0.63 ± 0.74 (0–2)	0.90 ± 0.97 (0–3)	NS		
Parity	0.38 ± 0.52 (0–1)	0.73 ± 0.84 (0–3)	NS		
Right endometrioma	5 (62.5%)	20 (47.6%)			
Left endometrioma	3 (37.5%)	22 (52.4%)	NS		
Diameter of largest cyst (cm)	4.75 ± 1.16 (3–7)	5.81 ± 1.99 (2–10)	NS	NS	
Multilocular cyst	1 (14.3%)	8 (19.1%)	NS		
Leiomyoma	4 (50.0%)	18 (42.9%)	NS		
Adenomyosis	1 (14.3%)	7 (16.7%)	NS		
Dysmenorrhea	5 (62.5%)	24 (58.5%)	NS		
Infertility	2 (25.0%)	2 (5.1%)	NS		
concurrent hysterectomy	0 (0.0%)	2 (4.8%)	NS		
r-ASRM score	48.3 ± 24.8 (26–90)	56.8 ± 29.2 (21–126)	NS	NS	
Adhesion score in the ipsilateral side	8.0 ± 26.8 (4–24)	12.1 ± 8.0 (0–32)	NS		
Adhesion score in the contralateral side	4.0 ± 2.1 (0–8)	5.4 ± 9.0 (0–32)	NS		
Adhesion score in the ipsilateral side ≥4	7 (87.5%)	38 (90.5%)	NS		
Adhesion score in the ipsilateral side ≥16	1 (12.5%)	20 (47.6%)	NS	NS	
Adhesion score in the contralateral side ≥4	7 (87.5%)	14 (33.3%)	*P* = 0.0067	P = 0.020	19.48 (1.59–237.72)
Adhesion score in the contralateral side ≥16	0 (0.0%)	5 (11.9%)	NS		
Pouch of Douglas obliteration	4 (50.0%)	29 (69.0%)	NS		
Postoperative follow-up period(month)	54.2 ± 11.2 (41–73)	44.5 ± 12.7 (15–69)	*P* = 0.0470		
Postoperative hormonal therapy	1 (12.5%)	9 (21.4%)	NS		
Oral contraceptives	0 (0.0%)	3 (7.1%)	NS		
Dienogest	1 (12.5%)	6 (14.3%)	NS		
Postoperative fertility treatment	1 (12.5%)	1 (2.4%)	NS		
Postoperative pregnancy	0 (0.0%)	0 (0.0%)	NS		
Postoperative parturition	0 (0.0%)	0 (0.0%)	NS		

**Fig. 1:**
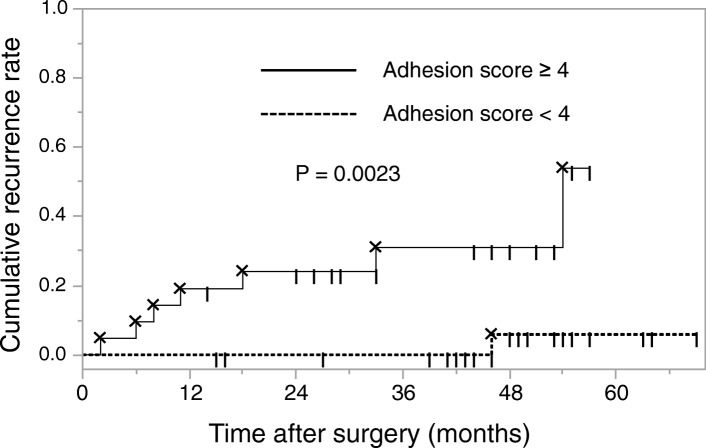
Kaplan-Meier analysis of endometrioma recurrence after unilateral salpingo-oophorectomy. There was a significant difference in recurrence in patients with a contralateral adhesion score ≥ 4 and those with contralateral adhesion score < 4 (*P* = 0.0023, log-rank test)

The cumulative recurrence rates by Kaplan-Meier analysis at 12, 24, 36, and 57 months postoperatively were 19.5, 24.1, 31.0, and 54.0%, respectively, in cases with an adhesion score ≥ 4, and 0.0, 0.0, 0.0, and 5.9%, respectively, in cases with an adhesion score < 4 (Fig. 1). Our comparison of cumulative recurrence rates revealed that the recurrence rate was significantly higher in cases with adhesion score ≥ 4 than in those with an adhesion score < 4 (*P* = 0.0023, log-rank analysis).

The cumulative recurrence rates by Kaplan-Meier analysis at 12, 24, 36, and 60 months postoperatively were 10.0, 10.0, 10.0, and 10.0%, respectively, in cases with postoperative hormonal therapy, and 7.5, 10.3, 13.6, and 27.3% in cases without postoperative hormonal therapy; this difference was not significant. The only case of endometrioma recurrence in the hormonal therapy group (Table 2) occurred in a patient who received low-dose dienogest (1 mg/day). After increasing her dienogest to the standard dose (2 mg/day), the size of the recurrent cyst decreased to less than 2 cm.

## Discussion

In this study of USO and conservative surgery performed for MRI-confirmed unilateral endometrioma, age, history of pregnancy, r-ASRM score, and the frequency of postoperative fertility treatment and hormonal therapy were different between patients who underwent USO and those who underwent cystectomy. Eight out of 50 patients who underwent USO for unilateral ovarian endometrioma had recurrence of endometrioma on the contralateral side. Univariate and logistic regression analyses of characteristics in groups with and without recurrence after USO revealed that a contralateral adhesion score ≥ 4 was a significant risk factor for endometrioma recurrence. Furthermore, there was a significant difference in the cumulative recurrence rate in patients with and without a contralateral side adhesion score ≥ 4. To our knowledge, this is the first study to evaluate the endometrioma recurrence rate and identify recurrence risk factors after USO for unilateral endometrioma.

Endometriosis is a chronic inflammatory disease that recurs after surgery and requires the maximum use of medical treatment to avoid repeated surgeries [14]. Patients diagnosed with endometriosis were reported to have a high risk of further surgery [15]. Although there is no report on recurrence rate after USO for unilateral ovarian endometrioma, we do encounter recurrent cases. Even after conservative surgery, the rates of recurrence of endometriosis on the ipsilateral and contralateral sides are reportedly similar [16]. Additionally, one study identified microscopic endometriosis, a phenomenon that could cause recurrence even after successful surgery [17]. Consequently, it is not surprising that endometriosis recurs on the contralateral side after USO.

With regard to patients’ background, age, parity, and the frequency of postoperative fertility treatment and pregnancy, there was significant difference between the USO and cystectomy groups, possibly because we did not offer USO to patients who wish to conceive. In addition, r-ASRM score and frequency of Douglas pouch obliteration were also significantly higher in the USO group, indicating that it was more severe compared to the cystectomy group. Furthermore, fewer USO group patients received postoperative hormonal treatment, which may have been withheld by clinicians under the impression that the post-USO recurrence rate is low due to the radical nature of the procedure, withheld such therapy. Since the patient backgrounds were widely varied, comparing the postoperative recurrence rates between the USO and cystectomy groups was inappropriate; however, on average, 16% in the USO group have relapsed in the postoperative follow-up period of 46 months; this indicates that the risk of postoperative recurrence also needs to be taken into account with USO.

Here, a contralateral side adhesion score ≥ 4 was identified as a recurrence risk factor after USO. However, we found no difference in the contralateral adhesion scores in cases with and without recurrence, and a contralateral adhesion score ≥ 16 was not a recurrence risk factor. Thus, while the presence of contralateral adhesions was significantly related to recurrence after USO, the severity of the adhesions was not. Regardless of the severity of adhesions, the fact that adhesion is formed itself is evidence that endometriosis extends to the contralateral side, and microscopic endometriosis may cause recurrence even after successful operation [17]. Therefore, we propose that the risk of recurrence should not be underestimated, even in patients with mild adhesions on the contralateral side.

In this study, all patients underwent preoperative MRI and received a transvaginal ultrasonography just prior to surgery, which confirmed the presence of a unilateral ovarian endometrioma and the absence of a contralateral endometrioma. In particular, MRI is the most powerful imaging technique in the diagnosis of ovarian endometrioma, with high sensitivity (90%), specificity (98%), and accuracy (96%) [18]. Therefore, patients in our study were unlikely to have a preoperatively undetected contralateral ovarian endometrioma.

We found no significant difference in recurrence rates with or without postoperative hormonal therapy. However, the sole case of recurrence in the hormonal therapy group might have occurred because the patient was receiving an inappropriate dienogest dose (half the usual dose). Indeed, when dienogest was increased to the standard dose, the recurrent cyst decreased in size. Furthermore, reports indicate that oral contraceptive [5, 11, 19] and dienogest [20, 21] administration lowered the endometrioma recurrence rate after conservative surgery. Thus, it seems theoretically possible that postoperative hormonal therapy could reduce the post-USO recurrence rate as well. Therefore, in cases of endometrioma accompanied by contralateral side adhesions, we propose prescribing postoperative hormonal therapy after USO.

Endometriosis is reportedly an ovarian cancer risk factor [3, 22–25]. Notably, ovarian endometrioma was implicated as a putative precursor lesion of endometrioid and clear cell ovarian cancer, and risk-reducing surgery has been recommended [3, 25]. Studies indicate that cystectomy does not reduce the risk of malignant transformation or ovarian cancer [26, 27]; however, the frequency of malignant transformation declined after USO [28, 29]. Therefore, USO could be an option that reduces the risk of malignant transformation.

Surgical menopause caused by bilateral salpingo-oophorectomy (BSO) has been correlated with an increased risk of cardiovascular disease, [30] dementia, [31] and Parkinsonism [32]. Furthermore, premenopausal USO reportedly reduces the age of menopause by 1.2 to 1.8 years, [33–35] although many, assuming that the remaining ovary compensates for the removed ovary, believe that USO is safer than BSO. However, whether USO affects the risk of cardiovascular events, dementia, and other diseases remains unknown. Future studies of USO and cystectomy outcomes are necessary to obtain evidence about postoperative recurrence rates, fertility preservation, surgical menopause, and potential for subsequent malignant transformation.

This study has several limitations. First, we performed a single-institution retrospective study; Therefore, our results require confirmation at other facilities. Second, patient characteristics differed between the USO and cystectomy groups, which could have caused bias. Therefore, in this study, we could not compare the recurrence rate between both groups. Third, we did not evaluate the recurrence of pain (e.g., dysmenorrhea and chronic pelvic pain), which is an important consideration when evaluating the effect of surgery in patients with endometriosis. A large-scale prospective study is required to overcome these limitations.

## Conclusion

To our knowledge, this is the first report on the recurrence rate and risk factors associated with recurrence after USO. In particular, the recurrence rate significantly increased in the presence of contralateral side adhesions. Thus, clinicians should be aware of the recurrence of endometriosis, even after USO, especially if contralateral adhesions are present. Our findings could improve the planning of USO and selection of patients for postoperative hormonal therapy.

## Abbreviations

BSO: Bilateral salpingo-oophorectomy
MRI: Magnetic resonance imaging
r-ASRM score: the revised American Society for Reproductive Medicine score
USO: Unilateral salpingo-oophorectomy
